# Membrane-Associated Heat Shock Proteins in Oncology: From Basic Research to New Theranostic Targets

**DOI:** 10.3390/cells9051263

**Published:** 2020-05-20

**Authors:** Maxim Shevtsov, Zsolt Balogi, William Khachatryan, Huile Gao, László Vígh, Gabriele Multhoff

**Affiliations:** 1 Center for Translational Cancer Research Technische Universität München (TranslaTUM), Radiation Immuno-Oncology group, Klinikum rechts der Isar, Einstein Str. 25, 81675 Munich, Germany; gabriele.multhoff@tum.de; 2 Institute of Cytology of the Russian Academy of Sciences (RAS), Tikhoretsky ave., 4, 194064 St. Petersburg, Russia; 3 Pavlov First Saint Petersburg State Medical University, L. Tolstogo str. 6/8, 197022 St. Petersburg, Russia; 4 Almazov National Medical Research Centre, Polenov Russian Scientific Research Institute of Neurosurgery, Mayakovskogo str. 12, 191104 St. Petersburg, Russia; wakhns@gmail.com; 5 National Center for Neurosurgery, Turan Ave., 34/1, Nur-Sultan 010000, Kazakhstan; 6 Far Eastern Federal University, Russky Island, 690000 Vladivostok, Russia; 7 Institute of Biochemistry and Medical Chemistry, Medical School, University of Pecs, Szigeti str. 12, 7624 Pecs, Hungary; zsolt.balogi@aok.pte.hu; 8 Key Laboratory of Drug-Targeting and Drug Delivery System of the Education Ministry, Sichuan Engineering Laboratory for Plant-Sourced Drug and Sichuan Research Center for Drug Precision Industrial Technology, West China School of Pharmacy, Sichuan University, Chengdu 610064, China; gaohuile@scu.edu.cn; 9 Institute of Biochemistry, Biological Research Centre, Temesvári krt. 62, 6726 Szeged, Hungary; vigh@lipidart.com

**Keywords:** heat shock proteins, HSP90, GRP96, HSP70, GRP78, HSP60, HSP40, HSP27, calreticulin, targeted diagnostics and therapy

## Abstract

Heat shock proteins (HSPs) constitute a large family of conserved proteins acting as molecular chaperones that play a key role in intracellular protein homeostasis, regulation of apoptosis, and protection from various stress factors (including hypoxia, thermal stress, oxidative stress). Apart from their intracellular localization, members of different HSP families such as small HSPs, HSP40, HSP60, HSP70 and HSP90 have been found to be localized on the plasma membrane of malignantly transformed cells. In the current article, the role of membrane-associated molecular chaperones in normal and tumor cells is comprehensively reviewed with implications of these proteins as plausible targets for cancer therapy and diagnostics.

## 1. Introduction

Heat shock protein (HSP) families consist of constitutive and stress-inducible members such as HSPB (small HSP), DNAJ (HSP40), HSPA (HSP70), HSPC (HSP90), HSPH (HSP110) and their related chaperokines HSPD/E (HSP60/HSP10) and CCT (TRiC) [1]. HSPs reside in the following three intracellular compartments and are also localized extracellularly where they fulfill various tasks: (1) cytosol (including various intracellular organelles (e.g., mitochondria); (2) nucleus; (3) and plasma membrane. Cytosolic chaperones play important roles in intracellular protein homeostasis including folding, unfolding and transport of denatured proteins and regulation of apoptosis. Upon stress such as hyperthermia, ionizing radiation, hypoxia, acidosis, and nutrient deprivation [2,3], their synthesis is rapidly upregulated by an activation of different heat shock factors (HSFs) in normal and tumor cells, although tumor cells per se exhibit elevated HSP levels already under physiological conditions due to their challenging microenvironment. A high HSP expression in various types of cancer cells is associated with tumor progression and resistance to anti-cancer therapies (including radio-/chemotherapies) [4]. Furthermore, upon stress (e.g., anoxia and hyperthermia) HSPs rapidly translocate into the nucleus, where they support their synthesis in an autocrine loop [5,6,7]. Extracellular HSPs were reported to play a role in both innate and adaptive anti-cancer immunity, implicating their possible application for development of immunotherapeutic approaches [8,9]. Thus, Hsp70 and Hsp90 proteins were shown to stimulate anti-tumor responses by facilitating cross-presentation of antigenic peptides via major histocompatibility complex (MHC) class I molecules, with subsequent induction of a CD8+ T cell-mediated immune response [9]. Furthermore, molecular chaperones of the HSP70 family can enhance cytolytic, migratory and proliferative capacities of natural killer (NK) cells even in the absence of immunogenic peptides [8,9].

Apart from their intracellular localization, various representatives of the major HSP families are reported to be expressed on the plasma membrane of cells [10,11,12,13,14]. Comprehensive profiling of the cell surface proteome of different tumor cell types (e.g., A549 lung adenocarcinoma, SH-SY5Y neuroblastoma, LoVo colon adenocarcinoma, Sup-B15 acute lymphoblastic leukemia, CX colon carcinoma and SKOV3 ovarian tumor cells) revealed the presence of numerous chaperones including HSP70, GRP75, GRP78, HSP60, HSP54, HSP27, and protein disulfide isomerase (PDI) on the plasma membrane [10,15]. Subsequent studies demonstrated the presence of other chaperones including HSP90, GRP96, HSP40, and calreticulin on tumor cell membranes [16,17,18]. Residents of the endoplasmatic reticulum (ER) (HSP47, GRP78, binding immunoglobulin protein (BiP), ERP57, PDI, GRP96, and calreticulin) gained relocalization signals (e.g., a KDEL sequence at the carboxy terminus) [17] or post-translational modification, which enables their transport to the plasma membrane (reviewed in [19]). However, for cytosolic HSPs (HSP70, HSP60, and HSP40), the exact mechanisms of protein transport, translocation through the membrane, and anchorage in the plasma membrane remain to be determined. In the current review, the role of cell surface-bound HSPs on tumor cells is discussed with respect to their possibility for development of novel diagnostic and therapeutic tools in oncology as well as their potential function in tumor progression and resistance to anti-cancer therapies.

## 2. HSP70 Family

**Figure 1:**
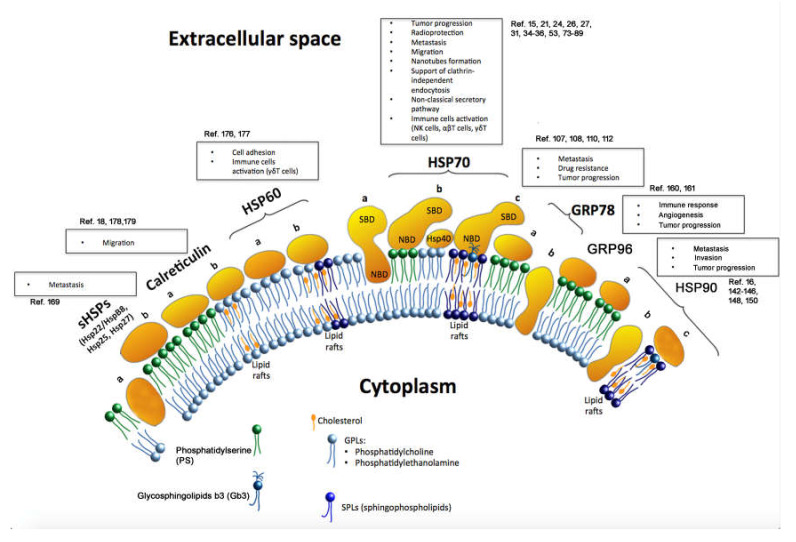
Membrane-associated heat shock proteins and their role in tumor pathogenesis.

The (1) inducible form of Hsp70 (HSPA1A) (but not the constitutive form, Hsc70) and (2) the ER-localized glucose-regulated protein 78 (GRP78) (HSPA5) (Figure 1) have been found to be localized on the plasma membrane of a variety of tumor cell types including primary glioblastomas [20], squamous cell carcinoma of the head and neck (HNSCC) and non-small-cell lung carcinoma (NSCLC) [21,22], human oral dysplasia and squamous cell carcinoma [23], colorectal and gastric cancer [24], pancreatic carcinoma [25], osteosarcoma [26], and acute myelogenous leukemia [25,27]. The transport of cytosolic Hsp70 to the plasma membrane occurs most likely via non-classical, vesicular mechanisms, since inhibitors of the post-Golgi membrane traffic by monensin or brefeldin A (BFA) do not impede the expression of membrane-bound Hsp70 (mHsp70) [28,29]. In a more recent study by Evdokimovskaya et al., application of BFA did not interfere with the secretion of Hsp70 (as well as Hsc70) by baby hamster kidney (BHK-21) cells, thus indicating a non-classical pathway of chaperone release [30].

Intriguingly, in patients with gastric and colon carcinomas mHsp70 expression correlated with an improved overall survival (OS), whereas a negative association was reported in squamous cell carcinoma and lower rectal cancer [24]. It is speculated that these contradictory results may be attributed to differences in the route of metastasis in these tumors or the complex role of Hsp70 in tumorigenesis. On the one hand, the hepatic route of metastasis of gastric and colon carcinomas might enable liver-residing CD56+ NK cells to deplete mHsp70+ tumor cells, and thereby might improve clinical outcome. On the other hand, mHsp70 was proposed to mediate a protective role against ionizing radiation by stabilizing lysosomal membranes (via enhancing the activity of acid spingomyelinase) [15,31,32], which protects tumor cells from lysosome-dependent cell death [33]. It has been shown that mHsp70 participates in non-classical secretory pathways [34,35] and facilitates clathrin-independent endocytosis [36]. An interaction of lysin-rich domains in the substrate-binding domain of Hsp70 directs Hsp70 monomers in an anti-parallel orientation [37], which facilitates dimerization, interaction with other co-chaperones (Hsp40, Hsp90, Hop) and HSP client proteins [38]. Presumably, post-translational modifications, especially in the substrate-binding domain (SBD), may also regulate Hsp70–lipid interactions, but further studies are required to prove this hypothesis [39,40,41,42,43]. Depletion of the C-terminal helical lid subdomain (ΔLSBD641 variant without the linker) also impairs Hsp70 oligomerization [37], which counteracts mHsp70-mediated facilitation of endocytosis [36]. In a B16/F10 mouse melanoma model, already 60 min following intravenous injection of rhHsp70-I^123^ the radiolabeled chaperone accumulated inside the tumor [44]. Cellular uptake of Hsp70 itself may also require its oligomerization on the cell surface [36,44,45,46,47,48,49].

Epitope mapping of the Hsp70-specific antibody cmHsp70.1 (aa 453–460), which detects cell surface bound Hsp70 on viable tumor cells with intact plasma membrane [50], revealed that the epitope of Hsp70 exposed on the plasma membrane of tumor cells is part of the oligomerization domain. Therefore, a 14-mer peptide TPP (aa 450–463) covering this region selectively binds to mHsp70+ tumor cells and can also become internalized. Based on these findings, a fluorescence and radiolabeled TPP peptide tracer has been developed that specifically targets mHsp70+ tumors in vitro and in tumor mouse models [51,52]. More recent studies demonstrated a role of mHsp70 in the formation of cell-to-cell connections via tunneling nanotubes (TNTs) in a 100 nm range, employing live-cell STED nanoscopy [53]. TNTs originate from cholesterol-rich microdomains, where mHsp70 co-localizes with the tumor-specific glycosphingolipid globoyltriaoslyceramide Gb3/CD77 [54]. 

Gb3, predominantly found in cholesterol-rich microdomains (CRMs), is overexpressed on the surface of tumor cells, as compared to corresponding normal cells [54]. Among other effects, a depletion of cholesterol by methyl-β-cyclodextrin reduces the amount of Gb3 concomitant with the amount of mHsp70 from the plasma membrane of tumor cells. Furthermore, in vitro experiments employing artificial liposomes consisting of PC/SM/Chol/Gb3 at a ratio of 17/45/33/5 confirmed a specific interaction of recombinant Hsp70 specifically with Gb3-containing vesicles [54]. Presumably, the interaction of Gb3 with the ATPase domain of Hsp70 in CRMs resembles the association of Hsp70 with 3’-sulfogalactolipid (SGL) [55]. Employing the truncated and mutagenized polymerase chain reaction products of the N-terminal Hsp70 fragments (NBD) including residues 318–387 (the base of the ATP-binding cleft) has shown that particularly Arg (342) and Phe (198) are crucial for binding of SGL [55]. Furthermore, time-resolved high-resolution AFM images as well as mutational analysis have proven the interaction of NBD with lipids [33,36,42,56]. In the study of Mahalka et al., it was proposed that Hsp70 can directly bind to membranes via insertion into the bilayers by the tryptophan residues Trp-580 in the SBD and Trp-90 in NBD [56].

Upon hypoxia stress or mild heat shock, mHsp70 co-localizes with the non-raft lipid component phosphatidylserine (PS) on the surface of tumor cells [57,58]. Presumably, the translocation of Hsp70 from the cytosol to the outer leaflet could be assisted by flipping of PS from the inner to the outer leaflet, although further experiments for elucidating this mechanism are required. A direct interaction of recombinant Hsp70 with PS was proven in artificial unilamellar phosphatidylcholine/phosphatidylserine (PC/PS) liposomes at different PC/PS ratios ranging from physiological ratios of 8:2 to 2:8, in which the highest interaction was observed in liposomes with the highest PS content. Charge-dependent, non-specific interactions of Hsp70 with lipids could be excluded since Hsp70 did not incorporate into phosphatidylcholine/phosphatidylglycerol (PC/PG, ratio 8:2) liposomes with identical charge. An interaction of exogenously administered Hsp70 with mHsp70 of stressed tumor cells can also occur by a protein–protein interaction via the extracellular-localized oligomerization domain of Hsp70 although further experiments are required to prove this hypothesis [36,42]. The binding of exogenous Hsp70 to PS at high concentrations (10–50 µg/mL) resulted in a concentration-dependent reduction in tumor cell viability (EC_50_ of Hsp70 = 55 μg/mL) and proliferation, which in turn enhanced the radiosensitization of hypoxic cells [57]. Screening the PS moieties with annexin V decreased the toxic effects of Hsp70 or Hsc70 that were added into the culture medium [59]. Subsequent experiments employing atomic force microscopy (AFM) have proven an association of recombinant Hsp70 with planar lipid monolayers at a dipalmitoylphosphatidylcholine/dipalmitoylphosphatidylserine (DPPC/DPPS) ratio of 80:20 mol% [60]. Presumably, an electrostatic Hsp70 interaction with lipids is essential for the initial docking with the membrane and that anchoring is driven by the alignment of protein domains with the dipalmitoyl chains of DPPS [60,61]. Further in vitro experiments confirmed an association of Hsp70 with PS and Gb3 [62,63,64].

Apart from heat shock and hypoxia several other stress factors including γ-irradiation and UV light [65,66,67], anti-inflammatory agents [68], cytostatic drugs (e.g., taxol, vincristinsulfate) [69], membrane-interacting alkyl-lysophospolipids [70], and HDAC inhibitors (antibiotic depsipeptide FR901228) [71] result in an upregulation of cytosolic and mHsp70 on tumor cells. Furthermore, exogenously administered recombinant human Hsp70 induces the relocation of its cytosolic form to the plasma membrane after internalization, and thereby increases the level of mHsp70 [48].

Membrane-bound Hsp70 also plays an important role in tumor immunosurveillance, serving as an antigen for the adaptive and innate immune system [9,29,72]. Hsp70-chaperoned tumor peptides presented on the plasma membrane of tumor cells are recognized by αβ and γδ T-lymphocytes [73,74]. As shown by Wei et al., a subsequent incubation of target cells with anti-Hsp70 antibody abrogated the cytotoxicity of OK432 (streptococcal preparation)-activated γδ T-lymphocytes [73]. In line with these findings, autologous polymorphonuclear neutrophils (PMNs) expressing mHsp70 are recognized and lysed by γδ T-lymphocytes, and thereby protect the host cells from inflammation-induced damage [75]. Furthermore, cells undergoing apoptotic cell death show an upregulated mHsp70 expression in the context with PS. Macrophages recognize PS on the outer leaflet as an “eat-me” signal [76,77,78].

Further studies have demonstrated that mHsp70 on tumor cells, even in the absence of HSP-chaperoned peptides, can be recognized by natural killer (NK) cells, particularly after ex vivo stimulation with Hsp70 peptide TKD and low-dose IL-2 [72,79,80]. Subsequent antibody blocking experiments suggest that the heterodimeric C-type lectin receptor CD94 in complex with NKG2C on NK cells serves as a potential receptor for mHsp70. A co-incubation of NK cells with TKD/IL-2 resulted in significant upregulation of the CD94 density on NK cells that was accompanied by an increased cytolytic activity mediated by an upregulated granzyme B production [81] against mHsp70+ tumor cells [82,83]. Preclinical studies employing human pancreatic (Colo357) and colon (CX2) carcinoma-bearing SCID/beige mice after intravenous injection of ex vivo TKD/IL-2-stimulated human NK cells have further proven the therapeutic potency of NK cells with respect to tumor growth control and reduction in liver metastasis [84,85]. Vice versa, the importance of mHsp70 for eliciting NK cell-based anti-tumor activity was demonstrated after treatment of tumor cells with vitamin A derivates, such as 13-*cis* retinoic acid (13-RA) or all-*trans* retinoic acid (ATRA), which are known to support redifferentiation of tumor towards normal cells concomitant with a loss in the mHsp70 expression [86]. A long-term co-incubation of tumor cells with these agents at non-toxic concentrations resulted in a significant decrease in the mHsp70 expression density, which in turn reduced the sensitivity of these redifferentiated cells to the cytolytic activity of NK cells [86].

In a phase I clinical trial, the safety, tolerability and feasibility of ex vivo TKD/IL-2-stimulated autologous NK cells were proven in 12 patients with advanced tumor stages (colorectal cancer, n = 11; NSCLC, n = 1) [87]. Based on these promising clinical data, a randomized multicenter phase II clinical trial (EudraCT 2008-002130-30) was started in patients with non-metastasized but locally advanced (IIIA and IIIB) NSCLC in combination with radiochemotherapy [88].

An interesting approach to restore tumor cell sensitivity towards cytolytic activity of NK cells was introduced by Sapozhnikov et al., employing the barnase:barstar pair for a targeted delivery of full-length Hsp70 or the 16 kDa C-terminal Hsp70 fragment to the plasma membrane [89]. In the first module, anti-HER2/neu mini-antibody conjugated with barnase was applied for a selective binding to the cell membrane of SKOV3 human ovarian adenocarcinoma and human BT-474 breast carcinoma cells. In a second step, the module barstar-Hsp70 (or its 16 kDa fragment) was attached to the first module, subsequently stimulating cytotoxic activity of NK cells against cancer cells, in vitro [89].

mHsp70 could be employed for the development of novel diagnostic and therapeutic (i.e., theranostic) Hsp70-targeting agents and could serve as a biomarker for detection and monitoring of tumors [90] or virally infected cells. Up-to-date radionuclide-, fluorescence-, nanoparticle-labeled mHsp70-targeted tools (including full recombinant Hsp70, monoclonal anti-Hsp70 antibodies, antibody Fab fragments, tumor penetrating peptide (TPP), granzyme B, and anticalines) have been successfully employed for visualization (MRI, PET, epifluorescence) and therapy in preclinical studies (Table 1). Thus, several studies demonstrated that mHsp70-targeted nanoparticles could be used for the detection and therapy of tumors [50,51,52,67,91]. In a recent study, functionalized nanoparticles with the serine protease granzyme B (GrB) (GrB-SPIONs) were used as a negative contrast enhancement agent for visualization of tumors by magnetic resonance imaging (MRI) and a pro-apoptotic therapeutic agent [91].

Another member of the HSP70 family, the ER-localized glucose-regulated protein 78 (GRP78), was also reported to be expressed on the membrane of tumor cells [10,92,93,94,95]. Due to the four hydrophobic domains capable of forming transmembrane helices localizing both the C- and N-terminal domains outside the membrane, GRP78 is expressed as a transmembrane protein [95]. In a recent study by Vig et al., it was demonstrated that GRP78 translocated to the membrane via the anterograde secretory pathway through Golgi complex, and DNAJC3 protein facilitated this process [96]. Membrane-bound GRP78 acts as a signaling receptor that, upon binding of its extracellular form sGRP78, initiated pro-apoptotic signaling cascades (increasing caspase 3/7 activity), which was accompanied by enhanced *Bax* and *Chop* mRNA expression [96]. Further studies have shown that GRP78 can also regulate the PI3K/Akt signaling [97,98]. Apart from direct embedding into the lipid bilayer, GRP78 can directly bind to transmembrane protein complexes and thereby interact with membranes [99].

Membrane-associated GRP78 was reported for hepatocellular carcinoma [100], prostate cancer [101,102], mammary carcinoma [103,104], lung [105,106] and gastric cancers [107,108].

mGRP78 has been shown to serve as a potential target for tumor-specific therapies (Table 1) [109]. Subsequent studies by Rauschert et al. demonstrated that apart from mGRP78 expressed on the cell membrane, its post-transcriptionally modified 82 kDa glycosylated isoform, termed GRP78^SAM-6^, is exposed particularly on the plasma membrane of a wide range of cancer types, but not on normal cells [109].

As reported by Papalas et al., expression of GRP78 in melanoma patients correlated with patient survival and invasive potential of the tumor [110]. Previously, it was demonstrated that GRP78 serves as a signaling receptor for activated α2-macroglubulin, microplasminogen, and plasminogen kringle 5, which functions as a receptor for angiogenic peptides. Furthermore, GRP78 is also involved in the MHC class I antigen presentation cascade [111,112]. Thus, binding of α2-macroglubulin to mGRP78 induces mitogenic signaling and tumor cell proliferation and increases metastatic spread [113,114]. Furthermore, it plays an important role for viral entry of dengue fever and coxsackie B virus. Subsequent studies by Arap et al. demonstrated that synthetic chimeric peptides designed from GRP78 binding motifs (i.e., WIFPWIQL and WDLAWMFRLPVG), fused to the programmed cell death-inducing sequence, can decrease tumor progression in preclinical models of breast and prostate cancer [115]. Application of monoclonal antibodies directed against the COOH-terminal domain of GRP78 also shows a pro-apoptotic activity (via upregulation of p53) in 1-LN and DY145 prostate cancer cells and A375 melanoma cells [116]. However, mGRP78 association was also reported for normal cells including macrophages, fibroblasts and endothelial cells, indicating possible off-target effects induced by anti-GRP78 therapies [112,117,118,119]. Indeed, in the study by Katanasaka et al., the authors demonstrated that GRP78-targeted WIFPWIQL-modified liposomes containing doxorubicin, efficiently bound to colon carcinoma cells and HUVEC endothelial cells [117]. To reduce unfavorable side effects of anti-GRP78 antibodies, a human monoclonal IgM antibody (SAM-6) derived from a gastric cancer patient was tested which is recognizing a cancer-specific GRP78–*O*-linked carbohydrate moiety [109]. Application of the SAM-6 IgM antibody resulted in a tumor-specific cell death via lipoptosis [120,121].

## 3. HSP90 Family

For the HSPC (HSP90) family, a membrane association was reported for two representatives: (1) Hsp90 (isoforms Hsp90α and Hsp90β), and (2) ER resident GRP96 (Figure 1, Table 2). In a study by Zhang et al. using surface plasmon resonance (SPR), it was shown that Hsp90 interacts with unsaturated phospholipids (i.e., POPS and POPG), and the observed affinity was higher when negatively charged lipids were involved (as compared to the zwitterionic lipids) [132]. Upon interaction with lipids, the α-helical structure of Hsp90 was increased, which may play a role for protein docking in the membrane. Addition of cholesterol to the lipid vesicles further enhanced the binding capacity of Hsp90. However, above a certain level of cholesterol (up to 50% of lipid composition) the association of Hsp90 was abrogated [132]. Presumably, localization of Hsp90 in the plasma membrane could increase its rigidity and integrity, which in turn protects cancer cells from various stress factors (including thermal stress) [132,133]. Indeed, for other HSPs (e.g., Hsp17), an association with the membrane resulted in a stabilization of the bilayer and a reduction in the membrane fluidity, thus providing thermotolerance and restoration of membrane functionality [11,12,134,135,136]. In a recent study by Li et al., it was demonstrated that Hsp90AA1 interacts with membrane phospholipids at high affinities not only via electrostatic interactions, but also by embedding its C-terminus into the bilipid layer which is accompanied with a conformational change of the protein (as shown by far-UV circular dichroism) [137]. Insertion of full-length Hsp90AA1 or its truncated form (Hsp90AA1-CTD) into membranes improved membrane integrity and induced thermotolerance in *Escherichia coli* (*E. coli*) [137]. A similar stabilizing role upon interaction with membrane phospholipids was described for Hsp90B1 from Anas platyrhnchos (ApHsp90B1) [138]. Apart from the C-terminus, the conserved amphipathic helix of Hsp90 was also found to play a role in the interaction of Hsp90 with membranes, and thereby promotes its exosomal release [139].

In addition to interactions with phospholipids, Hsp90 also has been found to be associated with lipid rafts in membranes [140]. Thus, the depletion of cholesterol results in the dissociation of Hsp70 and Hsp90 from lipid rafts [140].

An association of Hsp90 with the plasma membrane was shown for melanoma metastasis, but not for melanocytic lesions [141]. Additionally, mHsp90 upregulation, particularly the inducible isoform Hsp90α but not Hsp90β, on tumor cells was reported for fibrosarcoma HT-1080 and MDA-MB231 triple negative breast adenocarcinoma cells [16]. Hsp90α interacted with matrix metalloproteinase 2 (MMP2) in the extracellular space. Subsequent inhibition of Hsp90α employing anti-Hsp90 antibodies or scFvs significantly inhibited tumor cell invasion [16]. However, in another study employing HT-1080 and human A172 glioblastoma cells, the authors indicated that both Hsp90 isoforms, Hsp90α and Hsp90β, play a role for the motility of tumor cells. The cell surface heparan sulfate proteoglycans have been shown to play a role for the membrane expression of Hsp90 [142]. A decrease in sulfonation of heparan sulfates by heparinase I/III or heparin reduces the levels of both isoforms and subsequently inhibited cell motility [143]. An involvement of mHsp90 for tumor cell motility was further proven by small-molecule DMAG-N-oxide, a cell-impermeable 17DMAG-derived Hsp90 inhibitor [143]. The anti-invasive and anti-migratory activities of DMAG-N-oxide were demonstrated for different tumor cells types such as T24 bladder cancer, PC3M prostate cancer, and B16 melanoma cells in vitro at μM levels. Precise analysis revealed that mHsp90 might be involved in integrin signaling, which in turn influences focal adhesion. Subsequent in vivo studies showed that application of either DMAG-N-oxide or anti-Hsp90 antibody SPA830 reduced lung colonization after i.v. injection of B16 melanoma cells [143]. Employment of another monoclonal antibody 4C5 also reduced B16F10 melanoma metastasis in mice [144]. Furthermore, GA conjugated to cell-impermeable agarose beads could inhibit cell migration [16]. In the study by Cid et al., expression of mHsp90 was shown for the human neuroblastoma cells NB69 [145]. Furthermore, upregulation of mHsp90 was higher in undifferentiated spherical neuroblastoma cells as compared to more differentiated flattened cells [145]. Expression of mHsp90 was also shown to be important for the migration of neuronal cells, thus indicating a role of the protein in the development of the nervous system [146]. Additionally, it was reported that mononuclear cells obtained from patients with systemic lupus erythematosus also express mHsp90 [147]. Intriguingly, as shown by Li et al., extracellular Hsp90α can also regulate human fibroblast cell motility via the HIF-1 pathway and thereby influences wound healing in mice [148].

Another protein Grp96, the ER homologue of Hsp90, was shown to be associated with the plasma membrane and plays a role in the induction of immune responses [17]. mGrp96 expression was reported to be associated with tumor malignancy in certain tumor types [17,149,150]. mGrp96 can bind to the metalloproteinase domain with thrombospondin type 1 motifs 9 (pro-ADAMTS9), and a metalloprotease pro-a disintegrin-like domain that results in enhanced tumor progression and angiogenesis [151,152,153]. Intriguingly, various infections including *E. coli K1* and *Listeria monocytogenes* also upregulated the expression of mGrp96 [154,155,156]. Furthermore, mGrp96 expression was reported for murine immature thymocytes [157]. In a study by Hou et al., it was shown that mGrp96-targeted siRNA could significantly inhibit tumor growth and increase the overall survival of animals [158]. mGrp96 was shown to interact with HER2, thus facilitating the HER2 dimerization, with subsequent promotion of tumor cell proliferation. Inhibition of conformational Grp96 changes by an α-helix peptide decreases the HER2 dimerization, with subsequent inhibition of tumor cell growth, in vitro and in vivo [159]. Inhibition of mGrp96 by using anti-Grp96 monoclonal antibodies induces apoptosis and decreases tumor growth, in vivo [160].

## 4. Other Membrane-Associated HSPs

Among other HSPs, Hsp25 (murine homolog of human Hsp27) is known to be present on the plasma membrane of tumor cells (Figure 1) [161]. Previous studies demonstrated that large parts (particularly α-crystallin domain) of HSPB1 (Hsp27) and HSPB5 (αB-crystallin) are embedded in liposomes that contain a variety of phospholipids (i.e., POPS, POPC, and POPG) [162,163,164,165,166]. As shown by Bausero et al., high mHsp25 expression on 4T1 mammary carcinoma cells was associated with tumor progression and an increased metastatic spread into the lung in an o.t. mouse model [167]. Another member of the small heat shock protein family, Hsp22/HspB8, was shown to be expressed on the surface of human neuroblastoma SK-N-SH cells [168]. Subsequent in vitro studies, employing lipid vesicles containing phosphatidic acid, phosphatidylinositol or phosphatidylserine, demonstrated an association of Hsp22 with these lipids, which results in a conformational change of Hsp22 [168].

Hsp60 plasma membrane localization was shown for Daudi cells [169]. Application of two antibodies (N-20 and K-19) directed towards the amino- and carboxyl-terminus of mHsp60 revealed the presence of the full-length protein on the cell surface [169]. In another study, the authors demonstrated that mHsp60, like Hsp70, is associated with lipid rafts as well as with Golgi apparatus and exosomes [170]. Presumably, various stress-inducing factors could stimulate the expression of mHsp60. Indeed, in the study by Pfister et al., it was shown that upon heat shock, HUVECs start to present mHsp60 [171]. Subsequent atomic force microscopy (AFM) employing monoclonal antibody AbII-13 tethered to AFM tips confirmed the association of mHsp60 with plasma membrane in a patchy distribution pattern [171]. Another stress (i.e., acute injury) to the cardiac myocytes could also induce the expression of mHsp60 [172,173]. Presumably, mHsp60 plays a role in the activation of immune responses. Liver cells infected with intracellular bacterium *Listeria monocytogenes* exhibited high levels of mHsp60 [174]. Intriguingly, kinetic profiling of γδ T cell responses most closely matched that of mHsp60 expression in liver and tumors [175]. Further studies employing secondary human enterocyte-like Caco-2 cells demonstrated that heat shock (41 °C), TNFα, or *L. monocytogenes* infections (10^4^–10^6^ CFU/mL) increased the expression of mHsp60 and, as a result, enhances cell adhesion, but not invasion [175]. Subsequent application of shRNA reversed the adhesive properties of the cells.

Calreticulin, another ER-residing stress protein present on tumor cell membranes, was shown to induce migratory capacity in melanoma cells [18]. Application of anti-calreticulin antibodies significantly reduced laminin-dependent spreading of melanoma cells. As shown by Elton et al., calreticulin interacts with collagen receptors integrin α2β1 and glycoprotein VI in human platelets that could further support the hypothesis of calreticulin involvement in tumor cell spreading [176]. Calreticulin can bind in a Ca^2+^-dependent manner to phosphatidylserine (PS) with high affinity (*K_D_* = 1.5 × 10^−5^ M) [177,178]. Further studies demonstrated that calreticulin can be associated with lipid rafts in connection with ERP57 [179,180]. Thus, knockdown of ERP57 reduced the expression of calreticulin on the cell surface and subsequently reduces the phagocytic capacity of dendritic cells, which in turn decreases immunogenicity, in vivo [180].

Hsp40 (Hdj1) was shown to be associated with Hsp70 on the surface of CX+ colon and Colo+ pancreatic carcinoma cells [15]. Previously, it was shown that Hsp40 is secreted from cells via the Hsp/Hsc70-dependent exosome pathway [181]. Presumably interaction of the J-domain of Hsp40 with Hsp70 could explain the co-localization of the two proteins on the membrane. Other studies confirmed the expression of Hsp47 on scirrhous carcinoma of stomach and cervical carcinoma [182,183]. mHsp70+/mHsp40+ tumor cells demonstrated a high radioresistance [15]. However, as shown by Hebert et al., high levels of mHsp47 on the epidermoid carcinoma cell lines were associated with a low invasive capacity [184]. In contrast to other data, Yamamoto et al. showed that downregulation of Hsp47 by microRNA-29a (miR-29a) inhibited tumor cell migration and invasion in cervical squamous cell carcinoma [183].

Large parts of sHSPs (particularly α-crystallin domain) are embedded into lipid bilayers (a) or they can be present on the cell surface (b) [162,163,164,165,166,167]. Calreticulin was described to be associated with phosphatidylserine (a) or with lipid rafts (b) [18,176,177,178,179,180]. For Hsp60, the presence of full-length protein on the cell membrane (a) or in association with lipid rafts (b) was reported [169,170]. Hsp70 was shown to be embedded via its NBD in the outer layer of membranes (a), associated with the membrane surface (b) (presumably in close interaction with the J-domain of Hsp40 protein), and associated with glycosphingolipid Gb3/CD77 in cholesterol-rich microdomains (CRMs) (c) [33,36,54,56,57,58,59,60,61,62,63,64]. The ER-localized glucose-regulated protein 70 (GRP78) was shown to be present on the membrane surface (a) (including association with transmembrane protein complex, surface glycosylphosphatidylinositol-anchored proteins) or embedded into the lipid bilayer (b) [95,99]. Hsp90 was demonstrated to be present on the membrane surface (a), embedded into the membrane via its C-terminus (b) or can be associated with lipid rafts (c) [137,138,140]. Grp96, a homologue of Hsp90, was shown to be expressed on the membrane surface [17,149,150].

## 5. Conclusions

Apart from their important intracellular functions as molecular chaperones in regulating intracellular proteostasis, many HSPs have been shown to be expressed on the surface of various types of solid and hematological malignancies. An increased expression density of mHSPs is associated with tumor progression, resistance to anti-tumor therapies (e.g., radiochemotherapy), and an enhanced invasive and metastatic potential. Certain membrane-bound HSPs, including mHsp60, mHsp70 and their endoplasmatic analogues mGRP78, mGRP96, and membrane calreticulin, were demonstrated to induce innate and adaptive anti-cancer immunity. Exposure of the intracellular endoplasmatic chaperones on the plasma membrane of tumor and, in some cases, damaged normal cells, followed by subsequent activation of immunity represents a possible mechanism of immunologic surveillance for elimination of malignantly transformed or damaged cells. Some membrane-bound chaperones (e.g., Hsp70 and Hsp90) could also play a role in tumor cell association with extracellular matrices, thus influencing cell motility and invasion. Exposure of HSPs on the membrane is directly related to the composition of the lipid bilayer. Presumably, pharmacological modulation and/or modification of membrane lipid structures and microdomains could be further exploited in regards to chaperone expression, which in turn could be used for cancer therapies [185,186,187,188]. Expression of HSPs on malignant cells makes them a promising target for development of novel diagnostic and therapeutic approaches in oncology. Several preclinical and clinical studies listed in this review demonstrated their therapeutic activity in targeted therapies directed against membrane-bound HSPs.

## Figures and Tables

**Table 1:** Application of the membrane-associated Hsp70 and GRP78 for tumor theranostics.

mHsp70-Targeted Strategies							
mHsp70-Targeting Tool	Drug and Adjuvant Therapy	Application		Model	Injection Route	Results	Ref.
		Diagnostics	Therapy				
rhHsp70	rhHsp70-I^123^ rhHsp70-Alexa Fluor555	(1) Single-photon emission computer tomography (SPECT); (2) Confocal microscopy	N/A	(1) s.c. B16/F10 melanoma in C57Bl/6 mice, (2) o.t. C6 glioma in Wistar rats	i.v.	Accumulation of the rhHsp70-I^123^ in B16/F10 melanoma (24 h, KDN tumor/background = 3.43). Accumulation of rhHsp70-Alexa Fluor555 in C6 glioma after 24 h	[44]
rhHsp70	rhHsp70	N/A	+	o.t. C6 glioma in Wistar rats	i.t.	Increased OS. Enhanced infiltration of glioma with NK cells (Ly-6c+), T cells (CD3+, CD4+, CD8+). Elevated production of IFNγ and granzyme B	[45,47]
Hsp70	Hsp70-SPIONs	MRI	N/A	o.t. C6 glioma in Wistar rats	i.v.	Contrast MR enhancement	[122]
Hsp70	Hsp70-hydrogel + phloretin	N/A	+	s.c. B16 melanoma in C57Bl/6 mice	hydrogel + phloretin	Increased OS. Activation of innate and adaptive immune responses	[49]
Hsp70	Hsp70-hydrogel	N/A	+	s.c. B16F10 melanoma in C57Bl/6 mice	hydrogel	Reduced rate of tumor growth by 64%. Prolonged OS by 46%	[46]
rmHsp70	rmHsp70 + hyperthermia	N/A	+	s.c. B16 melanoma in C57Bl/6 mice	i.t.	Reduced tumor growth. Complete regression in 20% (2/10) of the mice. Induction of systemic anti-tumor immunity	[123]
Anti-Hsp72.000 MW antibody	Anti-Hsp70 antibody	N/A	+	in vitro Daudi cells (Burkitt B lymphoma), HIV+ H9 cells (CD4+ T cell lymphoma)	N/A	Enhanced ADCC against tumor cells	[124]
cmHsp70.1 antibodies	cmHsp70.1-miRNA (survivin)-NP	N/A	+	in vitro human U87 and LN229 glioblastoma cells	N/A	Enhanced radiation-induced increase in caspase 3/7 activity. Decrease in clonogenic cell survival	[125]
cmHsp70.1 antibodies	Cy5.5-cmHsp70.1	Intra-operative and near-infrared fluorescence imaging	N/A	i.p. and s.c. CT26 colon tumors in Balb/c mice	i.v.	Epifluorescence imaging of mHsp70+ CT26 tumors	[126]
cmHsp70.1 antibodies	cmHsp70.1 antibodies	N/A	+	s.c. CT26 tumors in Balb/c mice	i.v.	Induction of ADCC of mHsp70+ tumors. Inhibition of tumor growth. Increased OS	[50]
cmHsp70.1 antibodies	SPION-cmHsp70.1	MRI	N/A	o.t. C6 glioma in Wistar rats	i.v.	Contrast MR enhancement	[67]
cmHsp70.1 antibodies	cmHsp70.1-conjugated gold nanoparticles	Light microscopy	N/A	in vitro CT26, 4T1+, 4T1 cells	N/A	Specific accumulation of functionalized gold nanoparticles in mHsp70+ tumor cells	[127]
Hsp70-specific recombinant Fab fragment (Hsp70 Fab)	Cy5.5-Hsp70 Fab	Fluorescence microscopy	N/A	s.c. CT26 colon tumors in Balb/c mice	i.v.	Fluorescence imaging of mHsp70+ CT26 tumors	[128]
TPP peptide	TPP-PEG_24_-DFO [^89^Zr]	PET	N/A	(1) s.c. 4T1+ (mHsp70+) and 4T1wt breast carcinoma in Balb/c mice; (2) s.c. CT26 colon tumors in Balb/c mice	i.v.	Tumor-specific accumulation of the tracer in 4T1+ (6.2±1.1%ID/g), 4T1 (4.3±0.7%ID/g), and CT26 (2.6±0.6%ID/g) tumors	[52]
TKD peptide	TKD-modified doxorubicin (DOX)-loaded micelles (TKD-D-M)	N/A	+	s.c. MCF-7 breast tumors in Balb/c mice	i.v.	Accumulation of TKD-micelles in tumors. Inhibition of tumor cell proliferation	[129]
TPP peptide	Carboxy-fluorescein (CF)-labeled TPP	Confocal microscopy	N/A	in vitro MCF7 (82% mHsp70+), MDA-MB-231 (75% mHsp70+), T47D (29% mHsp70+), 4T1 and 4T1+ (>90% mHsp70+) cells	N/A	Specific binding and internalization by mHsp70+ tumor cells	[130]
TPP peptide	TPP [Cy5.5]	Epifluorescence microscopy	N/A	spontaneous pancreatic ductal adenocarcinoma (PDAC) mouse model, colitis-associated spontaneous tumor model in FvB mice, xenograft mouse model in SHO mice (HCT-116, CX-2, colon ca), (MCF-7, MDA-MB231, T-47D mammary ca), (Panc-1, MIA PaCa-2, COLO357, pancreatic CA), (H1339, A549; lung), (FaDu, Cal-33 head and neck ca), (HeLa, cervix ca)	i.v.	Epifluorescence imaging of mHsp70+ tumors	[51]
Granzyme B	Granzyme B	N/A	+	s.c. CT26 colon tumors in Balb/c mice	i.v.	Suppression of tumor growth	[81]
Granzyme B	(1) GrB-SPIONs; (2) GrB-Alexa688	(1) MRI; (2) Epifluorescence microscopy	+	o.t. C6 glioma in Wistar rats, o.t. human U87 glioma in NMRI nu/nu mice, o.t. mouse GL261 glioma in C57Bl/6 mice, o.t. human H1339 SCLC in NMRI nu/nu mice	i.v.	Contrast MR (GrB-SPIONs); Increased OS; Intraoperative tumor imaging (24 h, GrB-Alexa688)	[91]
Anticalin	^89^Zr-Anticalin	PET	N/A	s.c. FaDu tumors in immunodeficient CD1-*Foxn1^nu^* mice	i.v.	PET contrast enhancement in tumors	[131]
**mGRP78-Targeted Strategies**							
**mHsp70-Targeting Tool**	**Drug and Adjuvant Therapy**	**Application**		**Model**	**Administration**	**Results**	**Ref.**
		**Diagnostics**	**Therapy**				
Anti-GRP78 synthetic chimeric peptides (i.e., WIFPWIQL, WDLAWMFRLPVG)	Chimeric peptides fused with programmed cell death-inducing sequence (pro-apoptotic motif _D_(KLAKLAK)_2_)	N/A	+	DU145-derived human prostate cancer in nude mice, EF43-fgf4-derived isogenic tumors in Balb/c mice	i.v.	Suppression of tumor growth	[115]
Antibodies towards the COOH-terminal domain of GRP78	Antibodies towards the COOH-terminal domain of GRP78	N/A	+	in vitro 1-LN and DU145 prostate cancer cells, A375 melanoma cells	N/A	Anti-tumor pro-apoptotic activity due to an upregulation of p53	[116]
Anti-GRP78 targeting peptide WIFPWIQL	WIFPWIQL-modified liposomes containing doxorubicin	N/A	+	s.c. colon CT26 NL-17 carcinoma in Balb/c mice	i.v.	Suppression of tumor growth and increase in OS. Inhibition of tumor-induced angiogenesis	[117]
Human IgM antibody (SAM-6)	Human monoclonal IgM antibody (SAM-6)	N/A	+	s.c. mouse/human stomach carcinomas in NMRI nu/nu mice	i.p.	Tumor suppression	[121]
Human IgM antibody (SAM-6)	Human IgM antibody (SAM-6)	N/A	+	in vitro 23132/87 and BXPC-3, nasal septum squamous cell carcinoma RPMI-2650	N/A	Tumor cell death via lipoptosis	[120]

**Table 2:** Application of the membrane-associated Hsp90 and GRP96 for tumor theranostics.

mHsp90-Targeted Strategies							
mHsp90-Targeting Tool	Drug and Adjuvant Therapy	Application		Model	Administration	Results	Ref.
		Diagnostics	Therapy				
Anti-Hsp90 monoclonal antibody 1.5.1 and scFvs (IIIF1, IH5, IID3, IIC1, IIIG7, IIIC6)	Anti-Hsp90 antibody or scFvs	N/A	+	in vitro HT-1080 fibrosarcoma cells	N/A	Significant inhibition of tumor cell invasion	[16]
Anti-Hsp90 monoclonal antibody 4C5	Anti-Hsp90 monoclonal antibody 4C5	N/A	+	in vitro MDA-MB453 human breast carcinoma cells	N/A	Inhibition of cell invasion accompanied by altered actin dynamics. Disruption of surface HSP90/HER-2 inter-action that resulted in reduced HER-2 phosphorylation and impaired downstream kinase signaling	[146]
Anti-Hsp90 monoclonal antibody 4C5	Anti-Hsp90 monoclonal antibody 4C5	N/A	+	i.v. injection B16F10 melanoma in C57Bl/6 mice	i.p.	Significant inhibition of melanoma metastasis	[144]
(1) DMAG-N-oxide, cell-impermeable Hsp90 inhibitor; (2) Anti-Hsp90 antibody (SPA-830) targeting C-terminus	(1) DMAG-N-oxide, cell-impermeable Hsp90 inhibitor; (2) Anti-Hsp90 monoclonal antibody	N/A	+	in vitro T24, B16-luc, PC3M cells i.v. injection B16 melanoma cells in Nu/Nu mice s.c. B16 melanoma in Nu/Nu mice	i.v.	Significant in vitro inhibition of cell motility and invasion. Decrease in tumor cell colonization	[143]
**mGrp96-Targeted Strategies**							
**mHsp90-Targeting Tool**	**Drug and Adjuvant Therapy**	**Application**		**Model**	**Administration**	**Results**	**Ref.**
		**Diagnostics**	**Therapy**				
(1) Anti-mGrp96 siRNA; (2) Anti-mGrp96 antibody	(1) Anti-mGrp96 siRNA; (2) Anti-mGrp96 antibody	N/A	+	s.c. SK-Hep1 cells in nude mice, s.c. Huh7 cells in nude mice	i.p.	Inhibition of tumor growth and lung metastasis	[158]
Anti-mGrp96 antibody	Anti-mGrp96 antibody	N/A	+	s.c. SK-BR-3, T47D cells in Balb/c mice	i.v.	Induction of apoptosis and decrease in tumor growth	[160]
Anti-mGrp96 single α-helix peptide p37	Anti-mGrp96 single α-helix peptide p37	N/A	+	o.t. T47D, Bcap37 cells in nude mice	i.v.	Inhibition of tumor growth	[159]

## Abbreviations

ADP	adenosine diphosphate
AFM	atomic force microscopy
Akt	protein kinase B
ATP	adenosine triphosphate
BFA	brefeldin A
BiP	binding immunoglobulin protein
CRM	cholesterol-rich microdomain
DMAG	desmethoxy-17-*N*,*N*-dimethylaminoethylaminogeldanamycin
DNAJC3	DNAJ homolog subfamily C member 3
DPPC	dipalmitoylphosphatidylcholine
DPPS	dipalmitoylphosphatidylserine
ER	endoplasmatic reticulum
ERP57	endoplasmic reticulum (ER)-resident protein 57
GRP78	glucose regulated protein 78
GRP96	glucose regulated protein 96
HDAC	histone deacetylase
HIF-1α	hypoxia-inducible factor 1-alpha
HNSCC	head and neck squamous cell carcinoma
Hop	Hsp70-Hsp90 organizing protein
HSF	heat shock factor
HSP	heat shock proteins
HSP27	27 kDa heat shock proteins
HSP40	40 kDa heat shock proteins
HSP60	60 kDa heat shock proteins
HSP70	70 kDa heat shock proteins
HSP90	90 kDa heat shock proteins
IL-2	interleukin 2
MHC	major histocompatibility complex
mHsp70	membrane-bound Hsp70
MRI	magnetic resonance imaging
MMP2	matrix metalloproteinase 2
NBD	nucleotide binding domain
NK cells	natural killer cells
NSCLC	non-small-cell lung carcinoma
PC	phosphatidylcholine
PDI	protein disulfide-isomerase
PET	positron emission tomography
PI3K	phosphatidylinositol 3-kinase
PMNs	polymorphonuclear neutrophils
POPC	1-palmitoyl-2-oleoyl-sn-glycero-3-phosphocholine
POPG	1-palmitoyl-2-oleoyl-sn-glycero-3-phosphoglycerol
POPS	1-palmitoyl-2-oleoyl-sn-glycero-3-phospho-L-serine
PS	phosphatidylserine
SBD	substrate-binding domain
SGL	3’-sulfogalactolipid
SPIONs	superparamagnetic iron oxide nanoparticles
SPR	surface plasmon resonance
STED	stimulated emission depletion microscopy
TPP	tumor-penetrating peptide
